# The Role of Health Literacy and Social Networks in Arthritis Patients' Health Information-Seeking Behavior: A Qualitative Study

**DOI:** 10.1155/2012/397039

**Published:** 2012-09-10

**Authors:** Janette Ellis, Judy Mullan, Anthony Worsley, Nagesh Pai

**Affiliations:** ^1^Graduate School of Medicine, University of Wollongong, Wollongong, NSW 2522, Australia; ^2^School of Exercise and Nutrition Sciences, Deakin University, 221 Burwood Highway, Burwood, VIC 3125, Australia

## Abstract

*Background*. Patients engage in health information-seeking behaviour to maintain their wellbeing and to manage chronic diseases such as arthritis. Health literacy allows patients to understand available treatments and to critically appraise information they obtain from a wide range of sources. *Aims*. To explore how arthritis patients' health literacy affects engagement in arthritis-focused health information-seeking behaviour and the selection of sources of health information available through their informal social network. *Methods*. An exploratory, qualitative study consisting of one-on-one semi-structured interviews. Twenty participants with arthritis were recruited from community organizations. The interviews were designed to elicit participants' understanding about their arthritis and arthritis medication and to determine how the participants' health literacy informed selection of where they found information about their arthritis and pain medication. *Results*. Participants with low health literacy were less likely to be engaged with health information-seeking behaviour. Participants with intermediate health literacy were more likely to source arthritis-focused health information from newspapers, television, and within their informal social network. Those with high health literacy sourced information from the internet and specialist health sources and were providers of information within their informal social network. *Conclusion*. Health professionals need to be aware that levels of engagement in health information-seeking behaviour and sources of arthritis-focused health information may be related to their patients' health literacy.

## 1. Introduction

Health information-seeking behaviour has been identified as a key component of patient behaviour which assists in the psychosocial adjustment to illness [1]. Patients seek health information to manage their ongoing health, as well as to manage chronic disease [2]. Previous health information-seeking behaviour studies have focused on why patients engage in this behaviour, typically as a coping mechanism [1], from where they get their health information [3] and the kind of information they find [2, 4]. These studies often presume a level of patient health literacy sufficient for the patient to successfully navigate a range of activities, from taking medications safely to discussing their health concerns with health professionals [5]. Further, an individual's personal social network has been recognized as an important source of health information [3, 6] providing them with the support needed to manage a chronic illness, such as arthritis. 

Current chronic disease management models assume that the patient has a level of health literacy sufficient to understand the factors that may aggravate symptoms and the types of treatments that are effective in managing symptoms [7]. It is notable also that patients with low health literacy have been shown to have little understanding of the medications they are taking [8], which exacerbates the risk of poor health outcomes related to incorrect use of medications [9]. Further, an individual's health literacy may affect that individual's ability and desire to seek out health information [4, 10], and individuals with low health literacy are less likely to engage with written medicine information [10]. There are very few studies which have examined the impact of health literacy on arthritis medication management [5], so this is an appropriate area of research designed to better inform arthritis-focused patient management interventions. 

Patients who engage in health information-seeking behaviour tend to use sources of information that require active engagement, such as exposure to mass media (newspapers, television), the internet, and web-based social networks [3]. Most health information-seeking behaviour literature examines this behaviour generally and not specifically in arthritis patients. Informal social networks made up of family or friends [11] also provide a range of support, including informational support [12]. The sharing of health information through interpersonal communication has been found to be more prevalent among those with more complex informal social networks [13].

The aim of this paper is to undertake a pilot study using a constructivist paradigm [14] to investigate how arthritis patients' health literacy affected their engagement in arthritis-focused health information-seeking behaviour and how it directed their choice of health information source(s), importantly including their informal social network.

## 2. Method

The pilot study was exploratory, with a qualitative design using one-on-one semistructured interviews. A purposive selection strategy was utilized, targeting a convenience sample of community-dwelling adults taking medication prescribed by a health professional for the management of their arthritis and arthritic pain. Culturally and linguistically diverse (CALD) participants who could speak and understand English were also included in the study to maximize variance in health literacy and the range of experiences in managing chronic disease. Community groups in the Illawarra Region (New South Wales (NSW), Australia), which catered specifically to people with arthritis or to the elderly taking medication for arthritis, were targeted as part of the recruitment strategy. The community groups involved were the Illawarra Branch of the NSW Arthritis Foundation, the University of Wollongong Graduate School of Medicine patient volunteers program, and the South-Eastern Sydney and Illawarra Area Health Service Multicultural Health Outpatient Services. 

Following ethics approval by the University of Wollongong's Human Research Ethics Committee, the primary author attended a scheduled meeting for each of the community groups described above (at their usual premises), where she informed approximately 80 group members, in total, about the study at large and provided flyers with contact details for interested participants. These interested participants, then contacted the researcher who provided them with a participant information sheet and a participant consent form which was signed prior to the interview taking place at their usual community group meeting premises.

**Data Collection**

The participants completed a brief questionnaire (for demographic data) and an audit of their current medications to ensure that they were taking medications for arthritis pain. A one-on-one semistructured 30–60-minute interview then ensued (at their usual premises) which was recorded on digital audio equipment to allow for verbatim analysis of the discussion. The semistructured interview questions were designed to elicit the participants' experience in managing their arthritis medications and to describe which of these medications they felt were most effective in controlling their pain. The interview questions also asked the participants to comment on whether or not they discussed their pain medications with others, what they understood about their arthritis, and where they found information about their arthritis and arthritis pain medication.

**Data Analysis**

Digital audio recordings were transcribed and thematic analysis was conducted by the primary author utilising qualitative analysis software (N∗Vivo 8). Major themes and subthemes were inductively drawn from participant responses. The coding of participant responses was checked by an independent researcher. For the purposes of this paper, participant responses were categorized into a de facto measure of health literacy and to describe the extent and makeup of the participants' social networks, as described in the next two sections.

### 2.1. Measurement of Health Literacy

Health literacy was estimated and classified using a method adapted from the Field et al. [8] qualitative study into heart failure patients' understanding of medication (see Table 1). The health literacy analysis of the verbatim transcripts of the one-on-one semistructured interviews was independently conducted by two qualitative researchers who achieved a high level of inter-rater reliability (80% agreement). The classification of health literacy (adapted from Field et al. [8]) was based upon the participants' understanding of arthritis and its management, as well as emergent themes from the verbatim transcripts. Each respondent was assigned to one of these three levels by the primary author and another independent qualitative researcher (see Table 1). The level of engagement in arthritis-focused health information-seeking behaviour at each health literacy level was also examined from the verbatim interview transcripts.

### 2.2. Measurement of Social Network

Analysis of the transcribed interviews was used to determine the extent of participants' social networks, with participant responses categorized into three main themes—who was providing the support (formal, informal or other), the kind of support (informational, instrumental, appraisal, emotional), and the perceived quality of the support (positive or negative). Sources of informational support were examined and linked to each health literacy category to provide a de facto measure of health information sources.

## 3. Results and Discussion

### 3.1. Results

**Figure 1:**
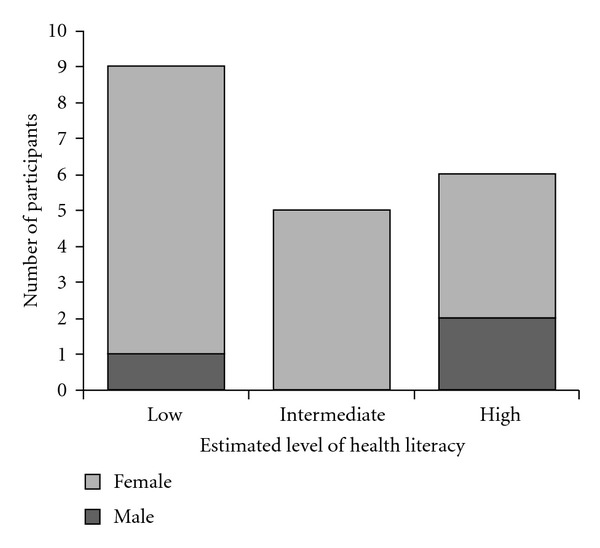
Number of male and female participants at each level of health literacy.

Twenty-one participants volunteered to take part in the study; however one participant was excluded for not fitting the selection criteria as she did not have arthritis.  Figure 1 indicates that more females than males participated in the study, and Table 2 shows that the majority of the participants were aged 75 years of age or less and from a CALD background, with equal numbers of participants being educated up to and below year 10, and above year 10. The CALD background participants were post-World War II emigrants from Central Europe (Germany *n* = 1), the Mediterranean (Italy *n* = 4; Greece *n* = 2), and a Balkan state (Macedonia *n* = 4). 

#### 3.1.1. Estimated Health Literacy Classifications

As shown in Table 2, just under half of the participants were estimated as demonstrating low health literacy. The remaining participants were almost evenly distributed between the intermediate and high categories of health literacy. The majority of those estimated as level 1 (low) and Level 2 (intermediate) health literacy were from CALD backgrounds, whereas the majority of the English-speaking background participants were estimated as Level 3 (high) health literacy. Similarly, the majority of participants who had been educated to year 10 and below were more likely to be represented in Level 1 health literacy and included many of the CALD participants. 

#### 3.1.2. Estimated Health Literacy and Level of Engagement in Health Information-Seeking Behaviour

Participants who were estimated as Level 1 (low) health literacy appeared to have little or no awareness or understanding of health information regarding their arthritis and/or pain medications. Notably, they showed little or no engagement in arthritis-focused health information-seeking behaviour, as demonstrated by the following comments

*Just as long as they [ease my pain], that's sufficient (Participant 4, female, ES background),*

*I am not questioning what the doctor, he is the expert, he is prescribing it for me (Participant 2, male, CALD background).*

Participants who were estimated as Level 2 (intermediate) health literacy appeared to understand the role pain medications played in the broader management of their arthritic pain and demonstrated some engagement in arthritis-focused health information-seeking behaviour. This was demonstrated by participants' awareness of health information, such as consumer medicine information, as evidenced by a comment such as

*I'm sort of fairly strict on that, you know,... and I'll sort of double check if it's not always on the packet when I come from the chemist...because sometimes you have to take it with food, sometimes you don't, you have to take it half an hour before food (Participant 3, female, ES background).*

Participants estimated as Level 3 (high) health literacy demonstrated more engagement with arthritis-focused health information-seeking behaviour. This was reflected by the higher level of technical detail used in these participants' conversations, such as this participant's response, in answer to a question about his understanding of his cervical spondylosis

*Oh, it's the narrowing of the channel in the top of my spine, which I understand to be caused by arthritis, osteoarthritis, the buildup of calcium in the bone, gradually narrowing the channel which affects my nervous system and is affecting my nervous system (Participant 12, male, ES background).*

#### 3.1.3. Sources of Health Information through Social Networks

The quality and complexity of information sources the participants engaged with appeared to be influenced by the participants' estimated level of health literacy. Furthermore, there appeared to be some differences in regard to the flow of health information within informal social networks.

While participants estimated as low health literacy did not report seeking health information outside their formal doctor/patient relationship, those estimated as intermediate health literacy were more likely to engage in general health information broadcast through television, radio, or newspapers. Some participants with estimated intermediate health literacy also reported that they looked for health information regarding their arthritis and its management on the internet, either through a general search or to follow-up programs they had heard on the radio or television. 

Those estimated as high health literacy were more likely to engage in specialized medical information, such as medical journals, health support organization reports, or their own medical records (such as X-ray reports), as well as consumer medicine information, as evidenced by this comment

*I belong to the Arthritis Foundation and I get their quarterly magazines (Participant 17, female, ES background).*

When it came to exchanging arthritis-focused health information within their informal social networks, the flow of information discussed by study participants also appeared to be linked to their estimated level of health literacy. Some participants from CALD backgrounds reported instances of seeking information about pain medications and supplements from friends in their informal social networks, such as

*I take this one because some of my friends say they good (Participant 29, female, CALD background).*

Those who discussed being recipients of advice from members of their informal social network tended to be in the estimated low and intermediate health literacy categories, whereas participants who stated that they gave advice to others in their informal social network tended to be in the estimated high health literacy category.

### 3.2. Discussion

The results of this study suggest that an individual's level of engagement with arthritis-focused health information-seeking behaviour is mediated by their level of health literacy and that they have access to a range of sources of information available through their informal social networks.

Those respondents who had been estimated to have high health literacy demonstrated the most engagement in arthritis-focused health information-seeking behaviour and appeared to inquire more about their arthritis and their pain medications. They reported seeking information from a variety of sources, mainly specialist medical texts (if the participant had access to them), health support organizations, and specialist sites on the internet. Patients such as these meet the criteria for the concordance effect that is the desired outcome of health literacy initiatives [8, 15–17], displaying characteristics of the *informed activated patient* exemplar of the Chronic Care Model [7]. One study examining the health attitudes, cognitions, and behaviours of individuals seeking health information found that *health-oriented* individuals utilized *active communication channels*, which required participant involvement in the critical analysis of health information [3]. Certainly, participants in this study with estimated high health literacy reported levels of personal and/or professional interest in health. This would imply that their enhanced ability to acquire and comprehend sources of quality arthritis health information facilitated their management of medications for their arthritis. 

In contrast, participants demonstrating estimated low health literacy described little or no engagement in arthritis-focused health information-seeking behaviour, accepting only the information received directly from a health professional. These participants did not describe seeking information from any other source and appeared to have fewer sources of informational support through their social network. A study into health information-seeking behaviour among social isolates found that those with limited social networks were less likely to seek information about their health [18]. However, it may be that those with low health literacy in this study do not have the capacity to seek information from other sources due to lower levels of education and, in the case of this study's sample, language differences, both barriers to health information sharing which have been identified in CALD populations in the United States [13]. The complexity of language in written and spoken arthritis health information [5], over and above the barriers of everyday written and spoken language, may also explain some of these results.

This study also suggests that the direction of the flow of health information within an individual's informal social network is affected by that individual's health literacy. Participants in this study who exhibited estimated high health literacy reported giving advice to others in their social network. Participants estimated as demonstrating intermediate and low health literacy reported receiving advice from those in their social network who had more understanding of health issues. This is consistent with another Australian study [19] which also found that those with high health literacy considered themselves sources of health information for those in their social network and that those with low health literacy looked to others in their informal social network for health information. More research into the flow of health information within social networks is necessary to confirm these exploratory findings.

Given that the majority of participants in this study who demonstrated estimated low health literacy were from CALD backgrounds, these results provide some insight into how patients with CALD backgrounds engage with health information. The value to patients with low health literacy of having access to those more confident in negotiating the health system is well established [13, 18, 20]. As there is limited formal evidence about this form of social support in CALD populations in Australia [21], more research into the characteristics of CALD populations' health information seeking behaviour is required.

The results of this study offer an insight into how the level of health literacy of patients managing their arthritis pain medication can influence their choice of health information sources. The study is important because if patients with a chronic illness, such as arthritis, are relying upon inadequate or inappropriate sources of health information, for example, from their informal social networks, they may be inadvertently misunderstanding their therapeutic regimen, which could then further exacerbate poor health outcomes and adverse drug events [9]. Health professionals need to be mindful that patients with a chronic illness, especially those with low health literacy, may not come to them to seek clarification about the health information given to them [19]. They also need to be especially mindful when treating patients from CALD backgrounds that they may not ask important questions about their care and/or therapeutic regimen because of their language limitations. In addition, based on the findings from this study, health professionals need to be aware that patients with high levels of health literacy are likely to seek further health information from other sources, which they are likely to share. Health professionals should therefore direct these patients to suitable and reputable health information resources to ensure that they are accessing and sharing good-quality health information.

**Limitations**

The small number of participants in our exploratory qualitative study limits the generalisations we can make about the role of health literacy and social networks in arthritis patients' health information seeking behaviour and prevents us from making any causal attributions. The very small proportion of males in the study precludes us from making any specific observations about similarities or differences between genders, and our study design assumed participants had some competent levels in speaking English. Further, the limited geographic spread of participants meant that many participants used the same health professionals (GPs or specialist rheumatologists) or belonged to the same community-based organizations. This may account for a social or cultural bias towards some behaviours or attitudes which might have influenced participant responses. The results of this study suggest that the research is worthy of further inquiry and needs to be validated in a larger study.

## 4. Conclusion 

Based on the results of this exploratory study, it appears that patients living with arthritis who have limited literacy skills and limited knowledge about their arthritis/arthritis pain medications perceive themselves to be managing their disease and medications according to what the doctor has prescribed. Importantly, however, it seems as though they are more likely to ask questions about their chronic condition and its treatment from their informal social networks rather than from their doctors. Further, this study highlights that patients with high literacy skills are more inclined to engage in arthritis-focused health information seeking behaviour beyond that provided by their doctors. These patients are also more likely to share this information and their knowledge among their informal social networks. Overall, therefore, even though much larger studies are required to confirm these findings, all health professionals should endeavour to encourage their patients with limited literacy skills, in particular those from CALD backgrounds, to ask questions to ensure that they are managing their chronic disease and pain medication safely and effectively. Health professionals should also ensure that their well-educated patients with high health literacy are sourcing information about their chronic disease and its management from good-quality, reputable sources, especially since it appears that many of them are likely to share their knowledge and understanding among others in their informal social networks. 

The results of this study have been presented in a poster presentation at the Australian Disease Management Association's 7th Annual National Conference, Canberra, August 2011, and the 10th National Emerging Researchers in Ageing Conference, Sydney, November 2011.

## Figures and Tables

**Table 1:** Classification of health literacy (adapted from Field et al. [8]).

Health literacy level	Classification criteria
Level 1—low health literacy	Little or no understanding of health information, use of nontechnical language, corresponding to Field et al.'s “Doing what I'm told”—for instance, participants who did not fully understand their arthritis or their arthritis pain medication and were not interested in further treatment details.

Level 2—intermediate health literacy	Some understanding of health information, use of a mix of technical and nontechnical language, corresponding to Field et al.'s “Leaving it up to your GP”—for instance, participants who described good relations with their GP and maintained that they received enough information about their arthritis and arthritis pain medication for their needs.

Level 3—high health literacy	Good to excellent understanding of health information, use of appropriate technical language, corresponding to Field et al.'s “Candidates for concordance”—for instance, participants who had a good to excellent level of understanding about their arthritis and arthritis pain medication and often sourced more information about their condition and its treatment.

**Table 2:** Number of participants at each level of health literacy, their age, cultural background, and education level.

Demographic	Estimated level of health literacy			Total
	Low	Intermediate	High	
Age				
≤75 years	4	5	5	14 (70%)
>75 years	5	0	1	6 (30%)
Cultural background				
English-speaking	1	2	6	9 (45%)
CALD	8	3	0	11 (55%)
Education level				
≤year 10	7	3	0	10 (50%)
>year 10	2	2	6	10 (50%)

Total	9 (45%)	5 (25%)	6 (30%)	20 (100%)
